# Efficacy and safety of intermittent preventive treatment for malaria in schoolchildren: a systematic review

**DOI:** 10.1186/s12936-015-0988-5

**Published:** 2015-11-14

**Authors:** Junior R. Matangila, Patrick Mitashi, Raquel A. Inocêncio da Luz, Pascal T. Lutumba, Jean-Pierre Van Geertruyden

**Affiliations:** Département de Médecine Tropicale, Faculté de Médecine, Université de Kinshasa, BP 747, Kinshasa, XI, Democratic Republic of the Congo; Epidemiology for Global Health Institute, University of Antwerp, Campus DrieEiken, Universiteitsplein 1, Wilrijk, 2610 Belgium

**Keywords:** Intermittent preventive treatment, Efficacy, Safety, Schoolchildren, Review

## Abstract

**Background:**

Intermittent preventive treatment (IPT) is a proven malaria control strategy in infants and pregnancy. School-aged children represent 26 % of the African population, and an increasing percentage of them are scholarized. Malaria is causing 50 % of deaths in this age group and malaria control efforts may shift the malaria burden to older age groups. Schools have been suggested as a platform for health interventions delivery (deworming, iron-folic acid, nutrients supplementation, (boost-)immunization) and as a possible delivery system for IPT in schoolchildren (IPTsc). However, the current evidence on the efficacy and safety of IPTsc is limited and the optimal therapeutic regimen remains controversial.

**Methods:**

A systematic search for studies reporting efficacy and safety of IPT in schoolchildren was conducted using PubMed, Web of Science, Clinicaltrials and WHO/ICTRP database, and abstracts from congresses with the following key words: intermittent, preventive treatment AND malaria OR *Plasmodium falciparum* AND schoolchildren NOT infant NOT pregnancy.

**Results:**

Five studies were identified. Most IPTsc regimes demonstrated substantial protection against malaria parasitaemia, with dihydroartemisinin-piperaquine (DP) given monthly having the highest protective effect (PE) (94 %; 95 % CI 93–96). Contrarily, SP did not provide any PE against parasitaemia. However, no IPT regimen provided a PE above 50 % in regard to anaemia, and highest protection was provided by SP+ amodiaquine (AQ) given four-monthly (50 %; 95 % CI 41–53). The best protection against clinical malaria was observed in children monthly treated with DP (97 %; 95 % CI 87–98). However, there was no protection when the drug was given three-monthly. No severe adverse events were associated with the drugs used for IPTsc.

**Conclusion:**

IPTsc may reduce the malaria-related burden in schoolchildren. However, more studies assessing efficacy of IPT in particular against malaria-related anaemia and clinical malaria in schoolchildren must be conducted.

## Background

Malaria is a major public health problem in sub-Saharan Africa (SSA), with 90 % of the burden occurring in African children [1–3]. To date, development and implementation of interventions for malaria prevention and control have been mainly directed towards well-known risk groups, such as pregnant women and children younger than 5 years old [4]. However, the impact of health factors on educational outcomes for schoolchildren is widely recognized, including by governments [5]. Previous research on the effect of malaria on children’s education showed that malaria is the leading cause of school absenteeism [6, 7] and asymptomatic malaria was likely to affect school performance [8]. In addition, malaria remains the biggest killer among school-aged children, causing 50 % of deaths in this age group in sub-Saharan Africa [9]. School-aged children represent 26 % of the African population, and an increasing percentage of them are scholarized. Since 2000, school enrolment increased by 52 % in sub-Saharan Africa [10]. Moreover, studies suggest that increasing control efforts and subsequent decline of the malaria burden as well as further progress towards malaria elimination will lead to a shift of at-risk population from under-five to older children groups, to which schoolchildren belong [11, 12]. Little research assesses adapted intervention tools for this vulnerable target group. Effective control measures could indeed reduce the burden of malaria in school-aged children. Sleeping under insecticide-treated nets can reduce overall child mortality [13]. Unfortunately, schoolchildren seem to be less likely to sleep under a bed net than those under-5 years old [14]. Active screening and treatment is another strategy under investigation. This approach may raise the question of the appropriate test to track asymptomatic malaria, since the latter is generally associated with relatively low parasite density [15]. Intermittent preventive treatment (IPT) may be another promising therapeutic strategy to prevent malaria and its related adverse outcomes in school-aged children [16]. This strategy has largely been studied in groups bearing the highest burden of the disease. In infants (IPTi), children younger than 5 years (IPTc) and pregnant women (IPTp), IPT schemes have been demonstrated to be protective against malaria and its related adverse outcomes [17–21]. However, only a handful of studies have focused on the potential benefits of IPT on school-aged children’s health (IPTsc). This review summarizes published results of this subject and it presents efficacy and safety data for all available therapeutic regimes in order to produce more information on their impact on asymptomatic and symptomatic parasitaemia, and haemoglobin concentration.

## Methods

### Search strategy and selection criteria

A single investigator (JMR) developed and conducted a systematic literature search for published IPTsc studies, on 28 April, 2014. Studies were identified using PubMed, Web of Science, Clinicaltrials [Clinicaltrials.gov and World Health Organization international clinical trials registry platform (WHO ICTRP) database] and abstracts from congresses. Combinations of the following search terms were used: intermittent, preventive treatment AND malaria OR *Plasmodium falciparum* AND schoolchildren NOT infant NOT pregnancy. Study eligibility was assessed in an unblinded manner. Studies had to be published articles written in English. Inclusion criteria were determined following the PICO format: (P) Participants: children of school age, resident in a malaria-endemic area; (I) Interventions: clinical trials evaluating the efficacy and safety of IPT; (C) Comparisons: the efficacy of available drug regimes compared to each other or with placebo; (O) Outcomes: the proportion of children with parasitaemia, anaemia and clinical malaria to provide the effect estimates (or sufficient data for calculation of an effect estimate), relative risk (RR), protective effect (PE), and their corresponding 95 % confidence interval (CI). If a selected study included more than one comparator or drug regimen, each comparison was regarded as a separate study. Studies which enrolled children younger than 5 years were included if data on children over 5 years were available for analysis. Studies evaluating chemoprophylaxis, screening and prompt treatment, as well as study protocol, were excluded.

### Data extraction

All the titles and abstracts were collected through the electronic search and filtered for potentially eligible articles. For each study, the following information was extracted: first author, publication year, year of study start and end, study design, randomization procedures, inclusion criteria, insecticide-treated net or bed-net use, local malaria transmission details of study groups, number of enrolled children, outcomes assessed and adverse events. Data from eligible studies were extracted based on the intention to-treat principle into a purpose-built database (Excel, Microsoft, 2010). This review follows the PRISMA guidelines [22].

## Results

### Study characteristics

**Fig. 1:**
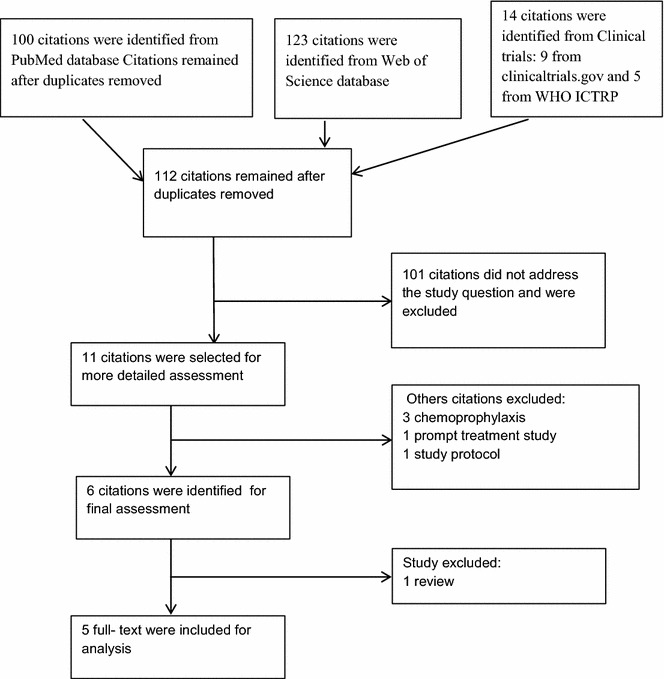
Flow chart study selection process of studies assessing efficacy of intermittent preventive treatment (2015)

A total of five trials assessing the efficacy of IPT for preventing asymptomatic and clinical malaria and anaemia in school-aged children were identified and included for analysis (Fig. 1) [23–27]. The trials were undertaken in Bondo in Kenya, in Kambila in Mali, Kollé in Mali, and in Tororo in Uganda. Malaria transmission patterns ranged from seasonal in the Malian sites over perennial with seasonal peaks in Kenya to hyperendemic in Tororo, Uganda. Usage of bed nets was low (<5–35.4 %) in all trials. All trials were randomized and placebo controlled, except for Kimbila (Mali), which was randomized and controlled. Two were double blinded, two open and one single blinded. Block randomization by individual was done in all trials except for Bondo (Kenya), which was cluster-randomized by schools.

IPT schedules differed between trials and were given every 5 months, four-monthly, three-monthly, bimonthly, monthly, or only at baseline (Table 1). All five trials had received ethics approval. The efficacy and safety of sulfadoxine-pyrimethamine (SP) was assessed in four trials: alone [23, 26], combined with amodiaquine (SP + AQ) [24], or with artesunate (SP + AS) [25]. Other combinations used were AQ + AS [25]. Dihydroartemisinin-piperaquine (DP) monotherapy was used in two trials in different schedules: at baseline, monthly and trimonthly [26, 27]. Safety and efficacy were assessed by passive and active clinical surveillance in all trials. The primary endpoint was the incidence of clinical malaria in three trials [23, 25, 27]. One trial’s primary endpoint was the risk of asymptomatic parasitaemia at 42 days [26] and the trial conducted in Bondo (Kenya) had the prevalence of anaemia as primary endpoint. However, three trials assessed the prevalence of anaemia or the increase in haemoglobin concentration as secondary endpoint [25–27]. The study duration differed between trials. Three trials evaluated the efficacy and safety of IPTsc on the basis of a 12 months’ follow-up [23, 24, 27], whereas in the remaining trials the follow-up period was of 5 months [25] and 42 days [26]. Thick blood films were stained and read by use of standard procedures for malaria parasite detection in all trials. Parasite density was calculated on the assumption of a mean of 8000 leucocytes/µL in two trials, or a mean of 7500 leucocytes/µl in Kambila (Mali) trial, whereas two trials did not specify the number of leucocytes for the parasite density calculation. The haemoglobin concentration was measured with a Hemocue^®^ photometer in all trials. Data on anaemia were reported in three of them [24, 25, 27]. Anaemia was defined as haemoglobin <11.0 g/dL [24, 25] and haemoglobin <11.5 g/dL for children aged 6–11 years and <12.0 g/dL for those aged 12–14 years [27]. The frequency and timing of outcome measurement varied greatly between studies. Malaria parasitaemia was assessed on days 7, 14, 28, and 42 [26] and monthly [25, 27]. The haemoglobin level was measure on day 42 [26], 6 weeks after the last treatment [24], every 3 and 5 months [27] and monthly [25]. Monitoring for clinical malaria was by active case detection [27], by active weekly follow-up visits and passive follow-up [23] and active monthly follow-up visits and passive follow-up [25].

**Table 1:** Characteristics of five trials of IPT in schoolchildren

	Clarke [24]	Dicko [23]	Barger [25]	Nankabirwa [26]	Nankabirwa [27]
Country and enrolment period	Kenya (Bondo), March 2005–March 2006	Mali (Kambila), July 2002–July 2003	Mali (Kollé), September 2007–January 2008	Uganda (Tororo), February 2008–July 2008	Uganda (Tororo), February 2011–February 2012
Pattern of transmission	Intense and perennial with seasonal peaks	Seasonal transmission	Seasonal transmission	Entomological inoculation rate in 2001–2002 was 562	high-intensity year-round, estimated entomological inoculation rate of 562
Bed net use	25.5 %	<5 %	Not available	27.7 %	35.4 %
Study design	Stratified, cluster randomized, double-blind, placebo-controlled	Randomized open controlled	Randomized open placebo-controlled trial	Randomized, single-blinded, placebo-controlled trial	Randomized, double-blind, placebo controlled trial
Number of children based on ITT	4916	262	296	780	740
Randomization	Stratified cluster	Individual	Individual	Individual	Individual
IPTsc drugs and timing of delivery	SP + AQ every 4 months	SP bimonthly	SP + AS and AQ + AS only at baseline	SP, SP + AQ and DP only at baseline	DP monthly and DP every 3 and 5 months
Age of study participants	5–18 years	6 months–10 years	6–13 years	8–12 years for girls and 8–14 years for boys	6–14 years
Follow-up duration	12 months	12 months	11 months	42 days	12 months
Primary endpoint	Prevalence of anaemia	Incidence of clinical malaria	Incidence of clinical malaria and risk of asymptomatic parasitaemia	Risk of asymptomatic parasitaemia at 42 days	Incidence of clinical malaria over 12 months.
Secondary endpoint	School performances	The in vivo response of *Plasmodium falciparum* to SP	Impact on haemoglobin concentration	Impact on haemoglobin	Parasite prevalence and anemia over 12 months
Control	Dual placebo	Passive surveillance	Placebo	Placebo	Placebo

*ITT* intention to treat analysis, *IPT* intermittent preventive treatment, *SP* Sulfadoxine-pyrimethamine, *SP* *+* *AQ* sulfadoxine-pyrimethamine plus amodiaquine, *SP* *+* *AS* sulfadoxine-pyrimethamine plus artesunate, *AQ* *+* *AS* amodiaquine plus artesunate, *DP* dihydroartemisinin-piperaquine

### Efficacy of IPTsc on malaria parasitaemia

**Fig. 2:**
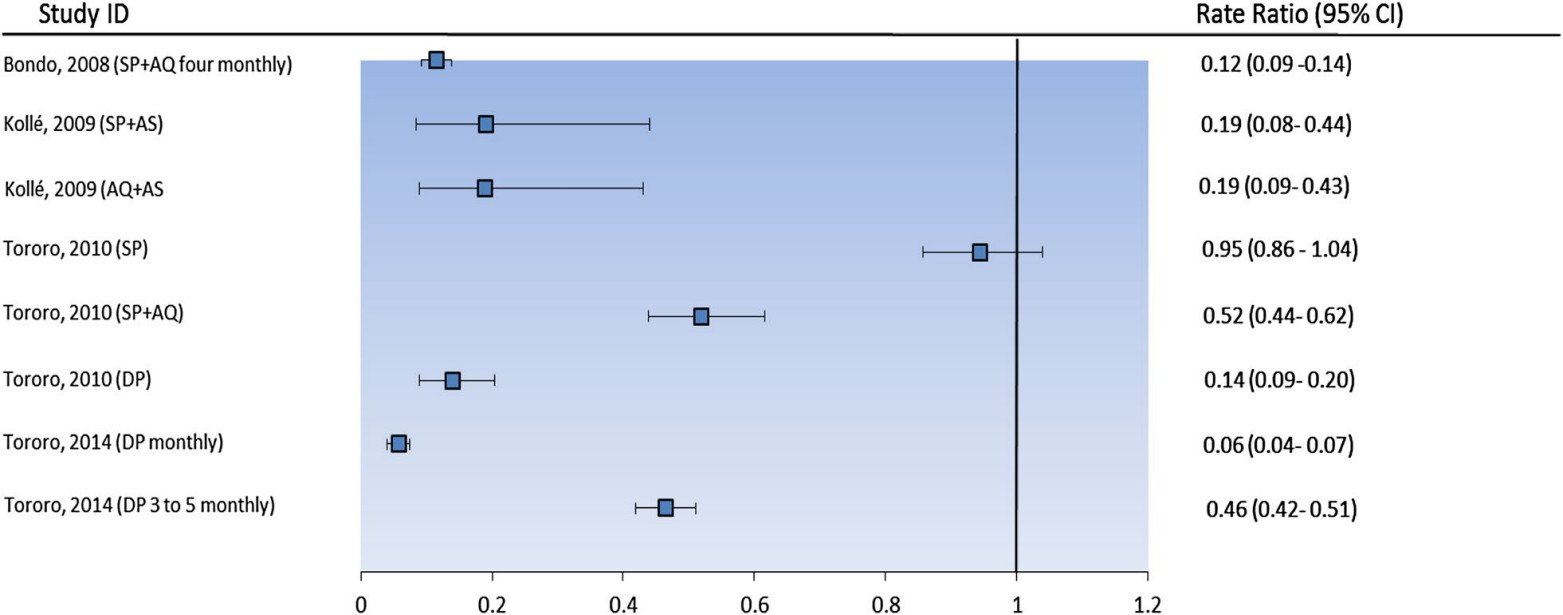
Effect of IPTsc on malaria parasitaemia during the intervention period. *RR* rate ratio, *SP* sulfadoxine-pyrimethamine, *SP* *+* *AQ* sulfadoxine-pyrimethamine plus amodiaquine, *SP* *+* *AS* sulfadoxine-pyrimethamine plus artesunate, *AQ* *+* *AS* amodiaquine plus artesunate, *DP* dihydroartemisinin-piperaquine, *CI* confidence interval

IPT with SP combined with AS and AQ + AS reduced significantly the risk of malaria parasitaemia. The prevalence of parasitaemia by treatment group was 6.6, 6.2 and 34.4 % for SP + AS, AQ + AS and vitamin C, respectively (P < 0.001) [25]. The risk of parasitaemia in DP and AQ + SP groups (11.7 and 44.3 %, respectively) was significantly lower than in those receiving SP (79.7 %) (risk difference AQ + SP vs placebo: 68.0 %; 95 % CI 60.6–75.4, p < 0.001; DP vs placebo: 35.4 %; 95 % CI 26.3–44.5, p < 0.001), and SP vs placebo: −4.9 %; 95 % CI −12.6 to 2.9, p = 0.2). DP was superior to AQ + SP (risk difference 32.6 (95 % CI 24.3–40.9); p < 0.001) [26]. SP plus AQ administered four-monthly reduced the prevalence of parasitaemia (SP + AQ: 4.6 %; 95 % CI 0.8–14.1) vs placebo: 39.7 %; 95 % CI 26.2–58.1 %, p < 0.0001) in Bondo, Kenya. The prevalence of parasitaemia was 2 % in DP monthly arm, 18 % in DP three-monthly and 38 % in control arm, in Uganda, 2014. The IPTsc with SP monotherapy in Tororo, Uganda did not demonstrate any PE against parasitaemia (0.05 %; 95 % CI −4 to 14). The PE of SP + AQ administered four-monthly was 88 % (95 % CI 86–90) in Bondo, Kenya, but this drug regimen demonstrated a lower PE (49 %; 95 % CI 38–56) in Tororo, Uganda. SP + AS and AQ + AS administered in Mali demonstrated similar PE (81 % (95 % CI 56–92) and 81 % (95 % CI 57–91), respectively. The PE of DP in Tororo in 2010 was 86 % (95 % CI 80–91) after 42 days’ follow-up. In 2014 the PE of DP given monthly in Tororo was the highest (94 %; 95 % CI 93–96), but this same drug regimen administered three-monthly in Tororo in 2014 had a significantly lower PE of 54 % (95 % CI 49–58) (Fig. 2; Table 2).

**Table 2:** Effect of IPTsc on malaria parasitaemia during the intervention period

	Study site	Study by drug regimen	No. of children	Prevalence of infection (%)	PE (95 % CI)
Clarke [24]	Bondo	SP + AQ (four-monthly)	2584	4.6	88 (86 to 90)
		Placebo	2294	39.7	–
Barger [25]	Kollé	SP + AS	91	6.6	81 (56 to 92)
		AQ + AS	97	6.2	81 (57 to 91)
		Placebo	96	34.4	–
Nankabirwa [26]	Tororo	SP	186	79.7	0.05 (−4 to 14)
		SP + AQ	200	43.3	49 (38 to 56)
		DP	198	11.7	86 (80 to 91)
		Placebo	196	84.6	–
Nankabirwa [27]	Tororo	DP (monthly)	2638^a^	2	94 (93 to 96)
		DP (three to five monthly)	2644^a^	18	54 (49 to 58)
		Placebo	2700^a^	38	–

*PE* protective effect, *No* number, *SP* sulfadoxine-pyrimethamine, *SP* *+* *AQ* sulfadoxine-pyrimethamine plus amodiaquine, *SP* *+* *AS* sulfadoxine-pyrimethamine plus artesunate, *AQ* *+* *AS* amodiaquine plus artesunate, *DP* dihydroartemisinin-piperaquine, *%* percentage

^a^Total tests

### Efficacy of IPTsc on anaemia

**Fig. 3:**
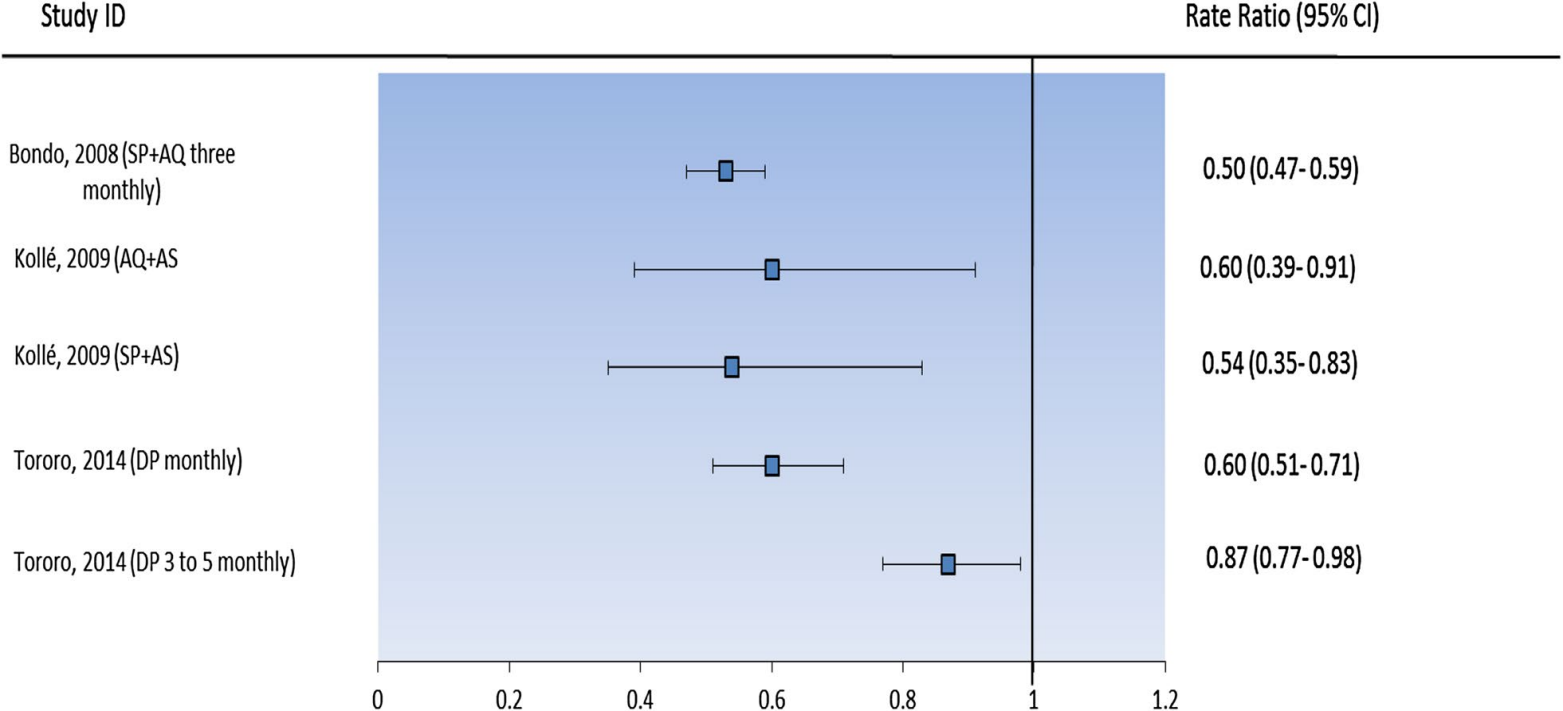
Effect of IPTsc on anaemia during the intervention period. *RR* rate ratio, *SP* sulfadoxine-pyrimethamine, *SP* *+* *AQ* sulfadoxine-pyrimethamine plus amodiaquine, *SP* *+* *AS* sulfadoxine-pyrimethamine plus artesunate, *AQ* *+* *AS* amodiaquine plus artesunate, *DP* dihydroartemisinin-piperaquine, *CI* confidence interval

Children treated with IPT were significantly less likely to be anaemic (SP + AS, 17.7 %; AQ + AS, 16.0 %; vitamin C, 29.6 %; P = 0.039) in Kollé, Mali. In Tororo, Uganda (2014), the prevalence of anaemia was significantly lower in the DP given monthly arm (12 %) but not the DP given three-monthly arm (17 %), compared with the placebo arm (20 %). SP + AQ (given four-monthly) reduced significantly the risk of anaemia (6.3 %), in Bondo, Kenya (SP + AQ: 6.3 % *vs* placebo: 12.6 %; p = 0.041). The PE effect of IPTsc on anaemia was generally lower in all trials that assessed this endpoint, and ranged from 14 to 50 %. SP + AQ (given three-monthly), SP + AS, AQ + AS and DP (administered monthly) demonstrated similar PE of IPTsc against anaemia in schoolchildren. The PE effect of DP given three monthly was the lowest (14 %; 95 % CI 2–23) (Table 3). The change in mean haemoglobin level did not differ between children who received SP + AS and AQ + AS, compared to those receiving placebo in Kollé, Mali. In Tororo as well, SP in 2010 and DP given three-monthly in 2014 did not increase the mean haemoglobin level compared to placebo. However, in the same trials held in Tororo, IPTsc with DP and SP + AQ (in 2010) increased the mean haemoglobin level (mean difference: 0.37 g/dL (95 % CI 0.18, 0.56) and 0.34 g/dL (95 % CI 0.15, 0.53), respectively) compared to baseline. Similarly, DP given monthly (in 2014) significantly increased the mean haemoglobin level compared to placebo group (1.20 vs 0.79 g/dL; P = 0.003). A high mean haemoglobin level was also observed in children treated with SP + AQ compared to those in placebo group (mean difference: 5.62 g/L (95 % CI 2.19–9.05) in Bondo, Kenya (Fig. 3).

**Table 3:** Effect of IPTsc on anaemia during the intervention period

	Study site	Drug regime	No of children	Prevalence of anaemia (%)	PE (95 % CI)
Clarke [24]	Bondo	SP + AQ (four-monthly)	2604	6.3	50 (41–53)
		Placebo	2302	12.6	–
Barger [25]	Kollé	SP + AS	96	17.7	40 (9–61)
		AQ + AS	100	16	46 (17–65)
		Control	98	29.6	–
Nankabirwa [27]	Tororo	DP (monthly)	717^a^	12	40 (29–49)
		DP (three and five monthly)	716^a^	17	14 (2–23)
		Placebo	736^a^	20	–

*PE* protective effect, *No* number, *SP* *+* *AQ* sulfadoxine-pyrimethamine plus amodiaquine, *SP* *+* *AS* sulfadoxine-pyrimethamine plus artesunate, *AQ* *+* *AS* amodiaquine plus artesunate, *DP* dihydroartemisinin-piperaquine, *%* percentage

^a^Total tests

### Efficacy of ITPsc on clinical malaria

**Fig. 4:**
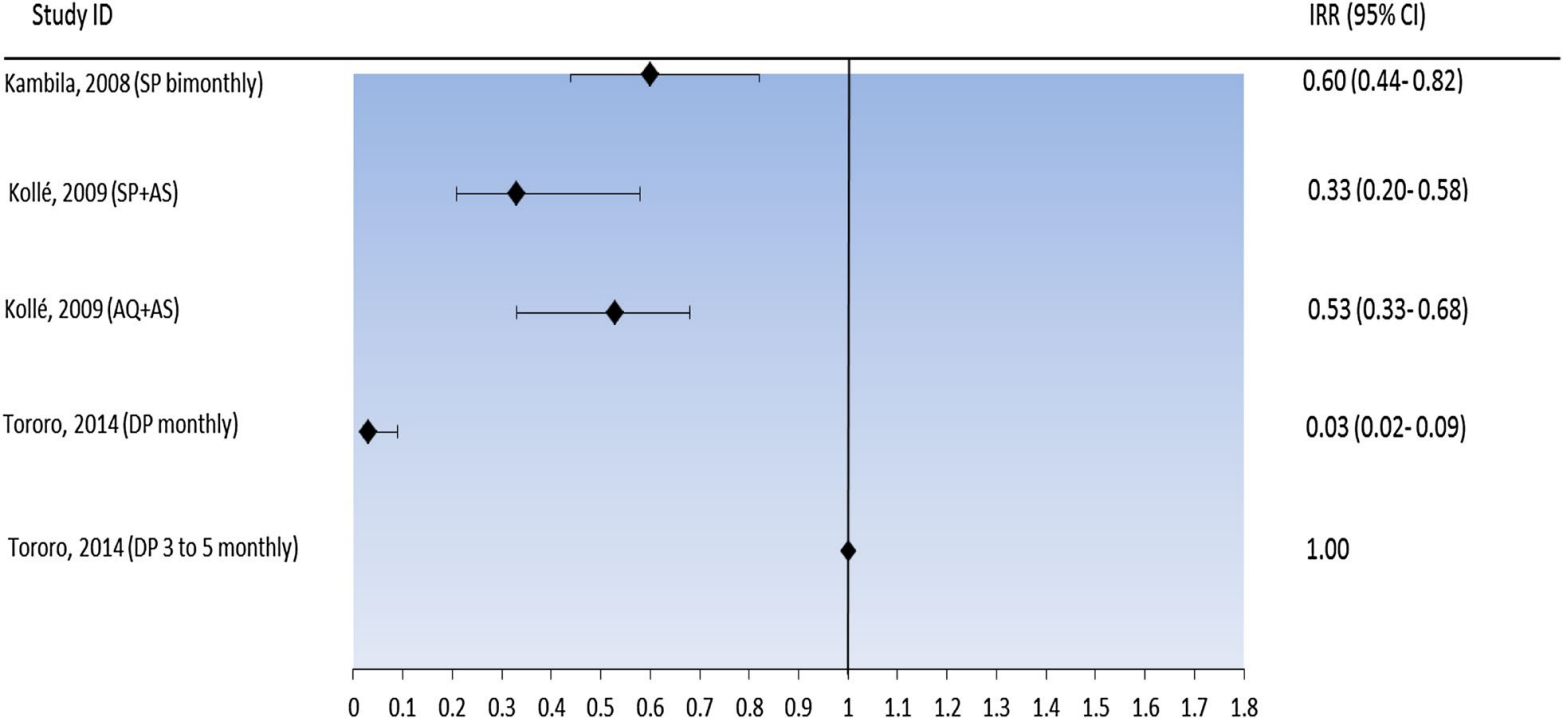
Effect of IPTsc on clinical malaria during the intervention period. *IRR* incidence rate ratio, *SP* sulfadoxine-pyrimethamine, *SP* *+* *AS* sulfadoxine-pyrimethamine plus artesunate, *AQ* *+* *AS* amodiaquine plus artesunate, *DP* dihydroartemisinin-piperaquine, *CI* confidence interval

The PE of IPTsc with SP given bimonthly against clinical malaria was 40.1 % (95 % CI 17.9–56.4) in Kambila, Mali. The PE of SP + AS in Mali was 67 % (95 % CI: 42–98). In the trial conducted in Kollé, Mali, AQ + AS had a PE of 47 % (95 % CI 32–67). In 2014 DP given monthly in Tororo had the highest PE (97 %; 95 % CI 87–98), however, this same drug regimen administered three- to five-monthly in Tororo in 2014 had a no PE (Fig. 4; Table 4).

**Table 4:** Effect of IPTsc on clinical malaria during the intervention period

	Study site	Drug regime	No of children	Incidence rate per 1000 PYAR	PE (95 % CI)
Dicko [23]	Kambila	SP (bimonthly)	…	2.7^a^	40.1 (17.9–56.4)
		Control	…	4.5^a^	–
Barger Barger [25]	Kollé	SP + AS	91	488	67 (42–98)
		AQ + AS	97	782	47 (32–67)
		Placebo	96	1463	–
Nankabirwa [27]	Tororo	DP (monthly)	236	0.01	97 (87–98)
		DP (three and five monthly)	234	0.34	0.00
		Placebo	243	0.34	–

*PE* protective effect, *No* number, *SP* sulfadoxine-pyrimethamine, *SP* *+* *AQ* sulfadoxine-pyrimethamine plus amodiaquine, *SP* *+* *AS* sulfadoxine-pyrimethamine plus artesunate, *DP* dihydroartemisinin-piperaquine, *…* data not available, *PYAR* person-year at risk

^a^5–10 years sub-group

### Safety outcome

Seven deaths occurred in the trial conducted in Bondo, Kenya, two in the IPT group and five in the placebo group. Cause of death could not be reliably ascertained in all cases. Twenty-three drug-related severe adverse events were observed during this study. Of these 23 adverse events, 19 occurred in the IPTsc goup (SP + AQ three-monthly) and four in the placebo group. The most commonly severe adverse events were: problems of balance, dizziness, feeling faint, nausea, or vomiting. One severe skin reaction was reported in the placebo group. In the study conducted in Tororo, Uganda, 2014, one death occurred in the IPT intervention group (DP three-monthly) due to acute lymphoblastic leukaemia. Fourteen severe adverse events were observed, but were judged unlikely to be associated with treatment. In almost all trials, mild or moderate adverse events were frequent in IPT group compared to placebo group. However, in the study conducted in Tororo (2014), mild or moderate adverse events including: fever, headache, nausea and vomiting were commonly associated with malaria, and were more frequent in placebo group than in the intervention arms. SP + AQ was significantly associated with vomiting in Tororo, 2010, and AQ + AS had the highest rate of headache in Kollé (Table 5).

**Table 5:** Safety of IPTsc during intervention period

Author (year)	Study site	Drug regime	No of children	Adverse events	Observation
Dicko [23]	Kambila	SP (bimonthly)	61	No severe adverse event	No data available
		Control	90		
Clarke [24]	Bondo	SP + AQ (four-monthly)	2604	19 (0.79 %) severe adverse events (problems of balance, dizziness, feeling faint, nausea or vomiting) 6 (0.23 %) adverse events graded as moderate 49 (1.9 %) mild events (nausea, headache, prurititis)	Mild events were more frequent among children receiving active drugs than among controls
		Placebo	2302	4 (0.17 %) severe adverse events (problems of balance, dizziness, feeling faint, nausea or vomiting and one severe skin reaction) 7 (0.30 %) moderate adverse events 33 (1.4 %) mild events (nausea, headache, prurititis)	
Barger [25]	Kollé	SP + AS	96	Most adverse events: headache, abdominal pain and respiratory symptoms.	–
		AQ + AS	100		
		Placebo	98		
Nankabirwa [26]	Tororo	SP	184	No severe adverse events. Only mild (92 %) and moderate (8 %), with no difference between treatment groups	–
		SP + AQ	197		
		DP	196		
		Placebo	192		
Nankabirwa [27]	Tororo	DP (monthly)	244	6 (2.5 %) severe adverse events	Mild events were more frequent in the placebo group than the intervention arms
		DP (three to five monthly)	248	1 (0.40 %) death (due to acute lymphoblastic leukaemia); 5 (2 %) severe adverse events	
		Placebo	248	3 (1.2 %) severe adverse events	

*No* number, *SP* sulfadoxine-pyrimethamine, *SP* *+* *AQ* sulfadoxine-pyrimethamine plus amodiaquine, *SP* *+* *AS* sulfadoxine-pyrimethamine plus artesunate, *AQ* *+* *AS* amodiaquine plus artesunate, *DP* dihydroartemisinin-piperaquine, *%* percentage

## Discussion

The aim of this review was to provide an overview of the effectiveness of IPT to prevent parasitaemia, clinical malaria and malaria-related anaemia in older children and to find out the most promising drug regimen for IPT in this specific population. The most appropriate drug regimen for IPTsc should provide protection against malaria-related anaemia and asymptomatic malaria parasitaemia, as these are the main features of malaria in this specific population. In this review, the studies used a wide variety of IPT regimes incorporating different drugs, dosages, timings, and numbers of IPT rounds.

The PE of IPT against malaria parasitaemia ranged from 49 to 94 % in the trials that assessed this endpoint. The highest PE was observed using DP monthly. SP + AQ given four-monthly, also demonstrated a high PE. SP monotherapy provided the lowest PE against malaria parasitaemia. This low efficacy of SP is not surprising and highlights the wide spread of resistance to this drug across Africa. The prevalence of 540E, one of the key SP resistance mediators, was found to be less than 50 % in Mali, whereas high prevalence of this marker (above 50 %) was observed in Uganda and Kenya [28]. This is in agreement with other findings from trials conducted in children younger than 5 years [29]. DP given monthly in Tororo, 2014, showed the highest PE against clinical malaria, but this drug given three-monthly did not show any PE against clinical malaria. These results are consistent with the theory stipulating that IPT protects by providing a period of post-treatment prophylaxis and that the length of this period of protection is determined by the pharmacodynamics of the drugs used [30].

The protective effect of IPTsc against malaria-related anaemia was relatively low in most trials that assessed this endpoint. DP given three- to five-monthly had the lowest PE against anaemia while DP given three- to five-monthly, or SP + AS and AQ + AS did not demonstrate any superiority in terms of mean change in haemoglobin levels compared to placebo. However, the combination of two drugs with long half-life (SP + AQ) in two studies [24, 26] increased the mean haemoglobin level compared to placebo. This relative low PE of ITPsc regimes on anaemia could be, at least partly, explained by the fact that haemoglobin or haematocrit continue to fall after treatment and may return to the normal level within 1 month from the start of malaria treatment. Moreover, malaria recrudescence has been found to delay haematological recovery [31]. Also, more importantly, the negative effect of malaria parasitaemia on heamoglobin level has been shown to decrease with age [32], suggesting that IPT would have a relatively low impact on malaria-related anaemia in schoolchildren. However a low PE of IPT against anaemia has also been observed in infants [33]. This adds more evidence on the expected efficacy of long half-life drug to protect against malaria parasitaemia recrudescence and thus against malarial anaemia. Given that malaria-related anaemia is multifactorial, involving haemolysis, iron deficiency due to impaired intestinal iron absorption, and red cell production failure (RCPF) [34, 35], the supplementation of ingested iron may rapidly replenish red blood cells (RBCs) and shorten haematological recovery after malaria infection has been cleared.

No deaths could be attributed to administration of drugs for IPTsc and observed severe adverse events occurred in both interventions and control groups. However, some adverse events judged mild or moderate were more frequent in intervention groups in one study.

Based on these findings, SP monotherapy should be considered the worst therapeutic regimen for IPTsc in area of high resistance to SP. However, SP has several benefits, including its low cost and proven safety. Moreover, studies have shown that SP could still be effective even in the presence of a high level of resistance in adults [36]. The underlying explanation could be that immunity increases with age and may modify the effect of anti-malarial drugs [37]. On the other hand, DP and SP + AQ appear to be the most promising drug regimen for IPTsc. However, given that the effect of IPT is mainly prophylactic, short-acting drugs such as artemesinins would be expected to provide little direct benefit in asymptomatic children and monthly administration of such drugs, though effective, may be challenging and costly. Moreover, the drug pressure in the context of IPT may lead to an increase of the selection of mutants resistant to artemisinin. Therefore, combination of SP with another long half-life such as piperaquine (PQ), AQ or mefloquine may be better option for investigation in clinical trials in schoolchildren. An additional advantage of combining two long half-life drugs is the reduction of risk of resistance to the two drugs as they have similar elimination half-lives [38]. IPT using long half-life drugs also appears to be more realistic in terms of frequency of drug administration, which can be bi- or three-monthly, and in children compliance.

This review suggests a need for harmonization in regard to the timing and frequency of drug administration as well as the time frame for drug evaluation in the context of IPT. IPT describes the administration of a drug at specified time intervals, allowing a very low concentration of drug (below the inhibitory level) between two treatments, with the aim of preventing mortality or morbidity. This should differ from chemoprophylaxis where the drug concentration is maintained above the level that inhibits parasitic growth [16]. A great variation in IPT drug timing and frequency of administration as well as the time frame for evaluation of IPT between studies has been observed, suggesting some overlaps between IPT and chemoprophylaxis. When a long-acting drug such as SP or piperaquine is used for IPT, a protective blood concentration may be sustained for several weeks, thus providing a period of chemoprophylaxis. Dicko et al. evaluated the efficacy of the SP administered every 2 months [23]. Given the long half-life of SP, this clinical trial should be considered evaluating chemoprophylaxis based on SP, rather than an IPT. In fact, a study conducted in the context of high resistance of SP reported that SP offered optimum protection until the fifth week after administration and that protection disappeared after the eighth week [30]. Similarly, the time frame for the evaluation of IPT should differ from that of chemoprophylaxis. An evaluation occurring when the drug concentration is optimal or above the minimum inhibitory concentration (MIC) would account for the effectiveness of chemoprophylaxis rather than an IPT.

Beside the choice of the appropriate drug regimen, one would anticipate the feasibility of this strategy. Schools have been suggested as a platform for health intervention delivery (deworming, iron-folic acid, nutrients supplementation, (boost-immunization). These interventions have been shown to improve not only children’s health and nutrition, but also their learning ability [39, 40]. Therefore, the increasing number of children of school-age and a higher number of these children attending a primary school [41] combined with the known impact of malaria on school-aged children’s health reasonably suggests that IPTsc could be part of the package of school health intervention, if this benefit is sufficiently proven.

The present study has a number of limitations. Since the search focused on the Pubmed database and articles published in English, there is a risk some other studies are missing. However, this is unlikely given the very limited number of trials conducted on IPTsc. Individual patient data were unavailable, therefore, the effect of baseline parasitaemia, age, ITN usage, or nutritional status on the efficacy of IPT could not be assessed. The high variability (heterogeneity in clinical, methodological and statistical aspects) across studies precludes a meta-analysis. Moreover, the small number of available studies could not allow a pooled analysis in sub-group regimes. Therefore, protective effect of regimes *vs* placebo or controls against clinical or asymptomatic malaria and anaemia were assessed without combined estimates.

## Conclusion

This systematic review found only five studies assessing the efficacy of IPTsc. Today, SP may not be a promising regimen for IPTsc in areas where resistance to this drug is high. Arteminisinin combination therapies (ACTs) (DP, SP + AS, AQ + AS) provided acceptable protective efficacy against clinical malaria, parasitaemia and anaemia. DP administered monthly demonstrated the highest PE. However, acceptability, feasibility and thus, effectiveness of a monthly administration of IPTsc is not known. Further, ACT is used for curative purposes. Combining at least two long half-life drugs, such as piperaquine plus SP may be, at present, the most promising option. More studies assessing efficacy of IPTsc in particular against malaria-related anaemia and clinical malaria must be conducted.

## Abbreviations

CI: confidence interval
DP: dihydroartemisinin-piperaquine
DRC: Democratic Republic of the Congo
IPT: intermittent preventive treatment
IPTsc: intermittent preventive treatment in schoolchildren
IRR: incidence rate ratio
PE: protective effect
PYAR: person years at risk
RCPF: red cell production failure
RR: rate ratio
SP: sulfadoxine-pyrimethamine
SP + AQ: sulfadoxine-pyrimethamine plus amodiaquine
SP + AS: sulfadoxine-pyrimethamine plus artesunate
AQ + AS: Amodiaquine plus artesunate
SSA: sub-Saharan Africa
DP: dihydroartemisinin-piperaquine
WHO: World Health Organization
ICTRP: International Clinical Trials Registry Platform
MIC: minimum inhibitory concentration
RBC: red blood cells
